# Lemborexant for insomnia in adults with psychiatric disorders: A 1‐week, open‐label study

**DOI:** 10.1002/pcn5.23

**Published:** 2022-06-30

**Authors:** Taro Kishi, Kenji Sakuma, Makoto Okuya, Nakao Iwata

**Affiliations:** ^1^ Department of Psychiatry Fujita Health University School of Medicine Toyoake Aichi Japan

## AIM

Most people with psychiatric disorders have insomnia.^1^, ^2^, ^3^ Our previous 1‐week, open‐label trial demonstrated that although suvorexant improved subjective total sleep time (sTST) and subjective time to sleep onset (sTSO) in adults with psychiatric disorders who did not receive any sleeping pills (ramelteon or Z‐drugs [zolpidem, zopiclone, or eszopiclone]) at baseline, it improved sTST but not sTSO in the individuals who had received those drugs at baseline.^4^ Our network meta‐analysis demonstrated that lemborexant, approved for insomnia treatment, had superior effects on sTSO compared to suvorexant.^5^ Lemborexant at 10 mg/day also outperformed suvorexant in sTST.^5^ Moreover, lemborexant did not differ from suvorexant in safety outcomes.^5^, ^6^ Here, we examined whether lemborexant had a benefit for the treatment of insomnia in adults with psychiatric disorders using a study protocol that was similar to our previous study on suvorexant.^4^

## METHODS

The study was conducted from March 2020 to March 2022 at Fujita Health University Hospital (UMIN000041321). The study protocol was similar to our previous study on suvorexant.^4^ Both female and male outpatients aged ≥ 20 years who experienced any of the following insomnia symptoms for ≥ 4 nights during the week before the study were included: sTST < 6 h, sTSO ≥ 30 min, and/or ≥2 wake episodes after sleep onset. The exclusion criteria were receiving any sleeping pills other than suvorexant, ramelteon, and the Z‐drugs at baseline; contraindications to lemborexant; addiction to psychostimulants or alcohol; pregnancy or breastfeeding; neurological or systemic diseases; and anyone deemed inappropriate to participate by the attending physician. The clinical trial was explained to the subjects, all of whom provided written informed consent, and was approved by the Ethics Committee of Fujita Health University (HM19‐407).

All subjects received a flexible dose of lemborexant for seven consecutive nights (the starting dose was 5 mg/day and the maximum dose was 10 mg/day). If suvorexant, ramelteon, or the Z‐drugs were provided at baseline, these were discontinued on study Day 1. Other psychotropic drugs were maintained at the same dose throughout the study.

The primary efficacy outcomes were sTST, sTSO, and subjective wake time after sleep onset (sWASO). Other efficacy outcomes included patients' sleep satisfaction level and self‐reported severity of mental illness. The subjects were assessed for all efficacy outcomes during the 2 days before they started the trial, after which baseline scores were calculated as the mean for the 2 days. All analyses were performed using data from the intent‐to‐treat population. Missing data were replaced using the last observation carried forward method. Given the small sample size, the Wilcoxon signed rank test was used to evaluate statistically significant differences in the efficacy scores between baseline and endpoint. To examine the impact of prior sleeping pill use on the results, subgroup analysis was performed to compare subjects who received any sleeping pills at baseline with those who did not. All statistics were performed using JMP software (JMP 14.2.0.; SAS Institute Inc.).

## RESULTS

This study included 56 adults—with a mean age of 46.82 ± 17.47 years and 18 (32.14%) males (Table S1)—of whom, nine (16.07%) discontinued the trial for the following reasons: adverse events (*n* = 5; dizziness [*n* = 2], agitation [*n* = 1], nightmares [*n* = 1], and sleep paralysis [*n* = 1]); inefficacy (*n* = 1); improvement in insomnia (*n* = 1); COVID‐19 infection (*n* = 1); and change in hospital (*n* = 1). The final mean lemborexant dose administered was 7.19 ± 2.48 mg/day.

**Table 1:** Efficacy results

	Total patients (*n* = 56)^a^			The subjects who received sleeping pills at baseline (*n* = 31)^b^			The subjects who did not receive sleeping pills at baseline (*n* = 25)^c^		
	Mean scores ± SD at baseline	Mean change scores ± SD	*p*‐value	Mean scores ± SD at baseline	Mean change scores ± SD	*p*‐value	Mean scores ± SD at baseline	Mean change scores ± SD	*p*‐value
Subjective total sleep time (h)	5.69 ± 2.20	1.27 ± 2.18	<0.0001	5.91 ± 2.35	1.08 ± 2.21	0.0148	5.42 ± 2.00	1.51 ± 2.16	0.0003
Subjective time to sleep onset (min)	74.06 ± 64.62	−42.07 ± 67.57	<0.0001	64.27 ± 50.10	−35.53 ± 49.05	<0.0001	86.20 ± 78.44	−50.17 ± 85.65	0.0051
Subjective wake time after sleep onset (min)	57.90 ± 73.36	−41.66 ± 73.49	<0.0001	56.37 ± 58.26	−41.60 ± 59.07	0.0002	59.80 ± 89.91	−41.74 ± 85.41	0.0226
Patients' sleep satisfaction level	3.42 ± 2.84	1.53 ± 2.77	<0.0001	4.19 ± 3.06	1.42 ± 3.28	0.0447	2.46 ± 2.25	1.73 ± 2.13	<0.0001
Severity of mental illness	4.07 ± 2.81	0.93 ± 2.15	0.0006	4.97 ± 2.97	0.81 ± 2.20	0.0162	2.96 ± 2.17	1.07 ± 2.12	0.0161

*Note*: A *p*‐value of < 0.05 was considered to denote statistical significance. Although we recognize that our results have multiple testing issues, uncorrected *p*‐values were presented because the sample size of our study was small.

The subjects' reported sleep satisfaction level and the severity of their psychiatric disorder: Sleep satisfaction was measured using a visual analog scale (VAS), with 0 = *very poor*, 5 = *average*, 10 = *excellent*. The severity of mental illness was measured using a VAS, with 0 = *extremely ill* and 10 = *no symptoms*.

^a^ Mean final lemborexant dose: 7.85 ± 1.70 mg/day.

^b^ Mean final lemborexant dose: 7.02 ± 2.45 mg/day.

^c^ Mean final lemborexant dose: 7.40 ± 2.55 mg/day.

Lemborexant significantly improved all efficacy outcome scores at the endpoint (Table 1). Both subgroups also demonstrated that lemborexant significantly improved all efficacy outcome scores at the endpoint (Table 1). No death was reported. The following adverse events were reported: at least one adverse event (*n* = 20), sleepiness (*n* = 4), fatigue (*n* = 5), nightmares (*n* = 2), headache (*n* = 4), dizziness (*n* = 2), dry mouth (*n* = 1), agitation (*n* = 1), and vomiting (*n* = 1).

## DISCUSSION

Lemborexant improved sTST, sTSO, sWASO, patients' sleep satisfaction level, and severity of psychiatric symptoms in adults with psychiatric disorders. For both subgroups, which divided individuals according to whether they received sleeping pills at baseline, we detected significant trends in all efficacy outcomes. However, although lemborexant had a risk for several adverse events, clinicians should monitor the individuals' health conditions. The main limitation of this study was the small sample size. The study included patients with different psychiatric disorders and medications, which cannot be ruled out as a possible confounding factor. Moreover, the study duration was short. Some participants might also perceive amelioration in insomnia symptoms as that in psychiatric symptoms. To obtain more robust evidence, a double‐blind, randomized, placebo‐controlled trial on lemborexant for adults with psychiatric disorders is required.

## AUTHOR CONTRIBUTIONS

Taro Kishi and Nakao Iwata contributed to the conception and study design. All authors contributed substantially to the analysis and interpretation of clinical data. Taro Kishi wrote the first draft of the article. All authors have contributed to and approved the final version of the manuscript. Nakao Iwata supervised this study.

## CONFLICT OF INTEREST

The authors declare no conflict of interest.

## DISCLOSURE STATEMENT

Interests for the past 3 years are as follows: Dr. Kishi has received speaker's honoraria from Sumitomo, Eisai, Janssen, Kyowa, Otsuka, Meiji, Takeda, and MSD, and research grants from Eisai, the Japanese Ministry of Health, Labour and Welfare, Grant‐in‐Aid for Scientific Research (C), and Fujita Health University School of Medicine. Dr. Sakuma has received speaker's honoraria from Eisai, Meiji, Otsuka, Kyowa, Takeda, and Sumitomo and has received a Fujita Health University School of Medicine Research Grant for Early‐Career Scientists and a Grant‐in‐Aid for Young Scientists (B). Dr. Okuya has received speaker's honoraria from Meiji, Otsuka, and Sumitomo and has received a Fujita Health University School of Medicine Research Grant for Early‐Career Scientists and a Grant‐in‐Aid for Young Scientists (B). Dr. Iwata has received speaker's honoraria from Astellas, Sumitomo, Eli Lilly, GlaxoSmithKline, Janssen, Yoshitomi, Otsuka, Meiji, Shionogi, Novartis, and Pfizer and research grants from Eisai, Takeda, Sumitomo, and Otsuka.

## ETHICS APPROVAL STATEMENT

The study was approved by the Ethics Committee of Fujita Health University (HM19‐407).

## PATIENT CONSENT STATEMENT

The clinical trial was described in detail to the subjects, all of whom provided written informed consent.

## CLINICAL TRIAL REGISTRATION

This study was registered in the UMIN Clinical Trials Registry as UMIN000041321.

## Supporting information

Supporting information.

## Data Availability

The entirety of the patients' data cannot be made publicly available as data sharing was not included in the consent.
