# Supplementary material for: Lemborexant for insomnia in adults with psychiatric disorders: A 1‐week, open‐label study
Source: PCN Rep. 2022 Jun 30;1(3):e23. doi: 10.1002/pcn5.23 (PMC11114363; doi:10.1002/pcn5.23)
Supplement: Supplementary file 1 — Supporting information. [file PCN5-1-e23-s001.docx]

Table S1. The patients’ characteristics

|  | Total patients (n = 56) | The subjects who received any sleeping pills at baseline (n = 31) | The subjects who did not receive taking any sleeping pills at baseline (n = 25) |
| --- | --- | --- | --- |
| Age (years, mean ± SD) | 46.82 ± 17.47 | 47.55 ± 18.03 | 45.92 ± 17.08 |
| Sex (%male) | 32.14 | 38.71 | 24.00 |
| Duration of illness (months, mean ± SD) | 75.22 ± 112.48 | 63.90 ± 88.87 | 89.83 ± 137.87 |
| Smoking status |  |  |  |
| Current smoker (%) | 21.43 | 16.13 | 28.00 |
| Past smoker (%) | 8.93 | 12.90 | 4.00 |
| Non smoker (%) | 69.64 | 70.97 | 68.00 |
| Drinking status |  |  |  |
| Everyday drinking (%) | 7.14 | 12.90 | 0.00 |
| Chance drinking (%) | 14.29 | 9.68 | 20.00 |
| Non drinker (%) | 78.57 | 77.42 | 80.00 |
| Primary diagnosis |  |  |  |
| Adjustment disorder (%) | 14.29 | 16.13 | 12.00 |
| Attention-deficit hyperactivity disorder (%) | 1.79 | 3.23 | 0.00 |
| Bipolar disorder (%) | 32.14 | 29.03 | 36.00 |
| Eating disorder (%) | 8.93 | 9.68 | 8.00 |
| Generalized anxiety disorder (%) | 3.57 | 6.45 | 0.00 |
| Major depressive disorder (%) | 21.43 | 19.35 | 24.00 |
| Panic disorder (%) | 1.79 | 0.00 | 4.00 |
| Schizophrenia (%) | 16.07 | 16.13 | 16.00 |
| Medication status |  |  |  |
| Antidepressants (%) | 41.07 | 48.39 | 32.00 |
| Antipsychotics (%) | 42.86 | 38.71 | 48.00 |
| Anxiolytics (%) | 7.14 | 3.23 | 12.00 |
| Lithium and anticonvulsant (%) | 23.21 | 32.26 | 12.00 |
| Sleeping pills^a^ (%) | 55.36 | 100.00 | 0.00 |
| Discontinuation rate (%) | 16.07 | 12.90 | 20.00 |

a. eszopiclone: 10 individuals [mean dose ± SD (mg/day) = 1.10 ± 0.32)], ramelteon: 2 individuals (8.00 ± 0.00), suvorexant: 17 individuals (18.53 ± 2.35), and zolpidem: 2 individuals (5.00 ± 0.00)
